# Managing nitrogen for sustainable crop production with reduced hydrological nitrogen losses under a winter wheat–summer maize rotation system: an eight-season field study

**DOI:** 10.3389/fpls.2023.1274943

**Published:** 2023-11-14

**Authors:** Li Wang, Lei Ma, Yan Li, Christoph-Martin Geilfus, Jianlin Wei, Fuli Zheng, Zhaohui Liu, Deshui Tan

**Affiliations:** ^1^ State Key Laboratory of Nutrient Use and Management, Institute of Agricultural Resources and Environment, Shandong Academy of Agricultural Sciences, Jinan, China; ^2^ Institute of Modern Agriculture on Yellow River Delta, Shandong Academy of Agricultural Sciences, Jinan, China; ^3^ State Key Laboratory of North China Crop Improvement and Regulation, Hebei Agricultural University, Baoding, China; ^4^ Department of Soil Science and Plant Nutrition, Hochschule Geisenheim University, Geisenheim, Germany

**Keywords:** nitrogen management, nitrogen leaching, nitrogen runoff, nitrogen use efficiency, wheat-maize rotation system

## Abstract

Excessive nitrogen (N) application in wheat–maize cropping systems was adjusted towards more sustainable practices to reduce hydrological N losses while maintaining crop yield. In comprehensive quantification of N management effects on crop yield, N use efficiency (NUE), hydrological N losses, and soil nitrate residual across eight seasons, we have added to growing evidence of strategies beneficial for sustainable crop production with lower hydrological N losses. The results show that adjusted N practices enhanced crop yield and NUE, as compared to farmer’s practices, but benefits varied with N rates and types. Optimized N treatment (OPT, 180 kg N ha^-1^ in both maize and wheat seasons) with or without straw returning produced the most crop yield. They increased maize yield by 5.5% and 7.3% and wheat yield by 6.2% and 3.2% on average, as compared to farmer’s practice with huge N application (FP, 345 kg N ha^−1^ and 240 kg N ha^−1^ in maize and wheat). Regulation of N release through amendment with controlled release urea at a rate of 144 kg N ha^−1^ crop^−1^ (CRU treatment) obtained 4.4% greater maize yield than FP, and sustained a similar wheat yield with less N input, resulting in the highest crop NUE. Additionally, CRU was most effective in mitigating hydrological N loss, with 39.5% and 45.5% less leachate N and 31.9% and 35.9% less runoff N loss than FP in maize and wheat seasons. Synthetic N input correlated significantly and positively with runoff and leachate N losses, indicating it was one of the dominant factors driving hydrological N losses. Moreover, compared to OPT, additional straw returning (STR) or substituting 20% of the nutrients by duck manure (DMS) further reduced runoff N discharges due to the fact that organic matter incorporation increased resilience to rainfall. N over-application in FP caused considerable nitrate accumulation in the 0–90-cm soil profile, while the adjusted N practices, i.e., OPT, STR, CRU, and DMS treatments effectively controlled it to a range of 79.6–92.9 kg N ha^−1^. This study suggests that efforts using optimized N treatment integrated with CRU or straw returning should be encouraged for sustainable crop production in this region.

## Introduction

Crop production must increase dramatically to meet the growing demand for food and biofuels projected for 2050 (Zhang et al., 2015). Over the past several decades, the improvement in global crop production has been associated with the increased use of nitrogen (N) fertilizer (Bodirsky et al., 2014; Kanakidou, 2019). However, on average, less than 50% of the nitrogen added to croplands globally is harvested as crop products (Ju et al., 2009). Inefficient use of N fertilizer by crops will result in substantial agricultural N losses, posing threats to human and ecosystem health. To boost crop yield with a lowered environmental cost, not only the use of high-potential crop cultivars but also efficient N management practices are required (Meng et al., 2016; Wang et al., 2019; Wang et al., 2020). Winter wheat–summer maize rotation is the most dominant cropping system in the North China Plain, one of China’s largest regions of agricultural importance (Sun et al., 2007; Zhang et al., 2018). Due to knowledge constraints and old habitats, N over-application and imbalanced use of P and K are common in farmers’ conventional practices. According to on-farm investigations, the N rates for maize and wheat production were as high as ca. 257 kg ha^−1^ and 325 kg ha^−1^ on average, while the estimated maize and wheat N uptake was only ca. 132 kg ha^−1^ and 160 kg ha^−1^, respectively (Cui et al., 2010; Chen et al., 2011), resulting in a high N surplus in the rotation system (Meng et al., 2016). To meet the dual challenges of increasing crop yield while mitigating adverse environmental impacts, many management strategies have been suggested to improve N use efficiency (NUE) (Qiu et al., 2012; Peng et al., 2017; Wang et al., 2019; Wang et al., 2020; Ren et al., 2023). However, the technological opportunity and socio-economic situation for NUE promotion differ regionally (Zhang et al., 2015). Suitable management practices have to be carefully adapted and adopted, taking advantage of local resources (Tan et al., 2013).

The Nansi Lake watershed is a part of the south-to-north water transfer scheme (Liu and Zheng, 2002), serving as an important water source in Shandong province, China. Excessive N fertilization in agricultural practices and abundant and concentrated precipitation have posed great risks to the water quality in this region. It was reported that ca. 40% of the total N entering the lake came from agricultural N loss through runoff and leachates (Tan et al., 2013). Following the government-guided shifts in agricultural management, local N management was converted towards more sustainable practices. Adjusting fertilizer amounts and types, such as balanced fertilization based on soil testing and the use of controlled-release urea (CRU), have been introduced, as these were believed to enhance crop NUE and minimize environmental pollution (He et al., 2009; He et al., 2013; Wang et al., 2018; Zheng et al., 2020). Moreover, huge amounts of crop straw and manure have been produced each year in this region, providing rich sources of organic N fertilizer that can be combined with synthetic N applications. Plenty of research has demonstrated improved cereal yield and NUE by the use of CRU (Li et al., 2018; Wang et al., 2018; Zheng et al., 2020) or co-application of organic and synthetic N fertilizers (Wei et al., 2016; Tang et al., 2019), because the N release synchronized better with plant N uptake. However, the effects of these management practices may vary with soil and weather conditions, cultivation regimes, CRU, and organic fertilizer types (Chivenge et al., 2010; Liu et al., 2021). A quantitative understanding of the practice effects on crop yield, NUE, and N losses is lacking in the Nansi Lake watershed.

To fill the knowledge gaps, an on-farm investigation was conducted over eight consecutive seasons to examine the effects of N management practices on mitigating hydrological N loss and sustaining crop yield under winter wheat–summer maize rotation. The specific objectives were to (1) quantify the crop yield and NUE as influenced by different N management practices and (2) explore the characteristics of runoff and leachate N losses to screen optimal N management practices.

## Materials and methods

### Study area

The experimental site is located in the Nansi Lake watershed (34°46′58″ N, 117°08′56″ E), Shandong province, North China, which has a temperate monsoon climate with an average annual precipitation of 550–720 mm and an average annual temperature of 14.4°C. The rainfall from June 2009 to June 2013 is shown in **Supplementary Figure S1**. The seasonal rainfall for maize was 359.1 mm, 524.6 mm, 479.9 mm, and 436.3 mm during the four growing seasons. In contrast, less rainfall occurred in wheat seasons, which were 260.1 mm, 72.7 mm, 222.3 mm, and 344.5 mm, respectively.

The soil in the experimental site is fluvo-aquic soil in an alluvial clay loam texture. The initial properties of the topsoil (0–20 cm) in 2009 were measured using standard chemical methods described by Sparks et al. (1996). They were as follows: pH 8.3, 8.87 g kg^−1^ organic carbon, 9.3 mg kg^−1^ available P, 140.8 mg kg^−1^ available K, 2.06 mg kg^−1^ nitrate-N (NO_3_^−^-N), and 1.69 mg kg^−1^ ammonium-N (NH_4_^+^-N). The dominant crop system in this region is winter wheat rotated with summer maize.

### Experimental design and management

A field experiment was carried out from June 2009 to June 2013. The experiment consisted of six treatments with three replicates that were arranged in a randomized complete block design. In total, 60-cm concrete levees bordered each plot with a size of 45 m^2^ to avoid water and fertilizer penetration. The treatments were (1) PK, with only P and K but no N fertilization; (2) farmers’ practice (FP) based on a survey of 15 households near the experimental sites; (3) optimized NPK treatment (OPT), designed by local experts based on soil nutrient analysis and crop nutrient demand; (4) CRU treatment, with the same P and K rates as in OPT, but N applied using resin-coated urea (42% N, Jinzhengda Ecological Engineering Co. Ltd., Shandong, China) at a reduced N rate by 20%; (5) duck manure supplement treatment (DMS), 20% of the total nutrients of the OPT replaced by the inputs from duck manure; and (6) straw returning treatment (STR), with the same fertilization rate as OPT plus straw returning. The detailed application rates of fertilizers for wheat and maize seasons are shown in **Table 1**.

**Table 1:** Fertilization rates (kg ha^−1^) for maize and wheat under different N management practices.

Treatment	Maize				Wheat			
	N	P_2_O_5_	K_2_O	Organic material	N	P_2_O_5_	K_2_O	Organic material
FP	345	0	0	0	240	172.5	0	0
PK	0	66	99	0	0	90	60	0
OPT	180	66	99	0	180	90	60	0
CRU	144	66	99	0	144	90	60	0
DMS	144	52.8	79.2	1,200	144	72	48	1,148
STR	180	66	99	6,000	180	90	60	7,500

FP, farmers’ fertilization practice; PK, P and K fertilizers only; OPT, optimized NPK fertilization; CRU, control-release N fertilization; DMS, optimized NPK fertilization with 20% of the total nutrients replaced by inputs from duck manure; STR, optimized NPK fertilization plus straw covering.

The basal fertilizers, including all P (triple superphosphate, 44% P_2_O_5_), K (potassium chloride, 60% K_2_O), CRU, duck manure, and 50% of normal N fertilizer (urea, 46% N), were broadcasted before soil plowing and leveling. The rest of N was top-dressed at the F3 and VT stages for wheat and maize. The N top-dressing was done by row application except in the FP treatment where N was broadcasted. In the STR treatment, wheat and maize straw at 6,000 and 7,500 kg ha^−1^ was spread evenly over the ground surface after harvest. Maize (cv. Zhongyu 9) was planted on 15 June and harvested on 7 October each year. Wheat (cv. Jimai 22) was planted on 12 October and harvested on 12 June the next year.

### Sampling and measurement

Leachate was sampled using an *in situ* collection plate (40 cm × 50 cm) inserted at a depth of 90 cm (Li et al., 2008). Soil surface runoff was collected by the runoff tank collection method (**Supplementary Figure S2**). Leachate and runoff samples were collected at V1, VT, R1, R3, and R6 stages for maize, and F2, F3, F4, F10, and F11 stages for wheat. Thereafter, leachate and runoff samples were transported immediately to the laboratory and stored below 0°C until analysis. Leachate and runoff volumes were recorded and the NO_3_^−^-N and NH_4_^+^-N concentrations were measured using an automatic flow analyzer. At harvest, all crops within each plot were manually harvested and threshed to determine the total yield after grains were air-dried. Maize and wheat samples were separated into grain and straw parts, and the N concentrations were measured after being digested with H_2_SO_4_-H_2_O_2_.

### Calculations

N uptake by maize and wheat plants was calculated by multiplying the biomass of grain and straw by the corresponding N concentrations.

The apparent fertilizer NUE was computed using the following equation:


(1) $$NUE=(T_{N}-T_{0})/F_{N}\times 100\%$$

Where *T_N_* was the plant N uptake in the plots receiving N fertilization; *T*_0_ was the plant N uptake in the treatment without N application (i.e., the PK treatment in the present study); *F_N_* was the fertilizer N amount.

To evaluate the sensibility of surface runoff to rainfall under different N management practices, the relationship between rainfall and runoff was analyzed with the linear regression model:


(2) y=a+bx

Where rainfall and runoff were used as the independent variable *x* and dependent variable *y*, respectively; the corresponding runoff sensibility to rainfall was defined as the first-order derivative of variable *y*, i.e., *b*.

The residual soil nitrate (RSN) in each soil layer was calculated according to Dai et al. (2016):


(3) $$RSN=T_{i}\times D_{i}\times C_{i}/10$$

Where *T_i_*, *D_i_*, and *C_i_* are the thickness, bulk density, and nitrate-N concentration of the corresponding soil layers.

### Statistical analyses

The effects of N management treatment, crop season, year, and their interactions on the measured parameters were tested via analysis of variance, with N management treatment, season as fixed factors, and year as a random factor. The means of different N management practices were separated by the least significant difference (LSD) test at the *p*< 0.05 probability level. The relationships between synthetic N input, runoff, and leachate N losses and between rainfall and runoff amount were analyzed with one-way regression analysis. Data were checked using the Shapiro–Wilk test for normal distribution before statistical analyses. All statistical tests were conducted with SPSS 20. Figures were plotted with SigmaPlot 12.0.

## Results

### Runoff and leachate amount

The high rainfall during maize seasons produced runoff and leachate at almost every stage of maize growth, with the exception of the V1 and R1 stages in 2012 (**Supplementary Figures S3**, **S4**). In contrast, runoff and leachate occurred less frequently in wheat seasons. For instance, only the F3 stage had runoff produced during the 2010–2011 wheat season.

N management practices resulted in considerable variation in the runoff amount (**Table 2**). In general, PK treatment was the highest in runoff, while STR was the lowest. The second-lowest runoff occurred in DMS. This treatment had on average 6.5%, 13.6%, and 9.2% less surface runoff than FP in maize, wheat seasons, and the whole rotations (*p*< 0.05), respectively. OPT and CRU did not vary with FP in runoff amounts in both maize and wheat seasons.

**Table 2:** Runoff and leachate amount during maize, wheat season, and the whole rotation under different N management practices.

Treatment	2009–2010			2010–2011			2011–2012			2012–2013		
	Maize	Wheat	Total	Maize	Wheat	Total	Maize	Wheat	Total	Maize	Wheat	Total
Runoff amount (mm)												
FP	48.9a	26.0b	74.9b	72.6b	12.3ab	84.9b	88.2b	45.0b	133.2b	67.7b	46.2b	113.9b
PK	49.8a	28.9a	78.6a	86.4a	13.1a	99.5a	99.2a	50.2a	149.4a	82.7a	51.3a	134.0a
OPT	49.1a	26.2b	75.2b	70.2b	12.1ab	82.3b	87.7b	44.4b	132.1b	65.8b	45.1b	110.9b
CRU	49.7a	26.9b	76.6b	74.5b	12.0ab	86.4b	89.8b	44.6b	134.5b	67.8b	48.0b	115.8b
DMS	47.7b	24.2c	71.9c	63.7c	11.3b	75.0c	83.9c	33.9c	117.8c	63.4c	39.5c	102.9c
STR	46.2c	19.8d	66.0d	56.6d	10.7c	67.2d	79.1d	28.8d	107.8d	58.9d	35.0d	93.9d
Leachate amount (mm)												
FP	103.6c	49.2c	149.8c	123.5c	25.7b	148.7c	143.8c	51.4c	195.2c	145.0c	71.3c	216.3c
PK	95.3d	45.4d	140.7d	111.2d	23.5c	134.7d	128.3d	46.5d	174.8d	136.3d	67.8d	204.1d
OPT	104.5c	48.5c	153.0c	121.5c	26.9b	147.9c	142.8c	49.5c	192.3c	140.6c	73.1c	213.7c
CRU	96.2d	46.3d	142.5d	122.9c	23.6c	146.5c	145.4c	48.2c	193.6c	142.1c	69.5cd	211.6c
DMS	109.7b	55.6b	165.3b	129.6a	27.5b	157.1a	153.9b	55.5b	209.4b	153.4b	80.9b	234.3b
STR	118.8a	61.7a	180.5a	132.7a	30.0a	162.7a	159.3a	59.5a	218.8a	157.5a	86.7a	244.2a

FP, farmers’ fertilization practice; PK, P and K fertilizers only; OPT, optimized NPK fertilization; CRU, control-release N fertilization; DMS, optimized NPK fertilization with 20% of the total nutrients replaced by inputs from duck manure; STR, optimized NPK fertilization plus straw covering. Means within a column followed by the same letter are not significantly different at p< 0.05 level, LSD.

Moreover, the runoff amount increased linearly with rainfall, as shown in **Table 3**. In line with its highest runoff amounts, the PK treatment showed significantly higher *b* values than other treatments in both the maize and wheat seasons, indicating a greater runoff sensibility to rainfall. STR and DMS significantly reduced the runoff sensibility to rainfall, as compared to FP treatment.

**Table 3:** Regression analysis (*y* = *a* + *bx*) showing the sensibility of runoff (*y*) to rainfall (*x*) during maize and wheat seasons under different N management practices.

Treatment	Crop season	*a*	*b*	*R* ^2^	*p*-value
FP	Maize	2.752 ± 2.485a	0.133 ± 0.025b	0.604	< 0.001
PK		2.469 ± 2.793a	0.160 ± 0.028a	0.638	< 0.001
OPT		2.621 ± 2.291a	0.131 ± 0.023b	0.638	< 0.001
CRU		2.641 ± 2.155a	0.137 ± 0.022b	0.683	< 0.001
DMS		2.061 ± 1.952a	0.130 ± 0.020b	0.703	< 0.001
STR		1.832 ± 1.912a	0.122 ± 0.019c	0.685	< 0.001
FP	Wheat	0.558 ± 1.697a	0.131 ± 0.026b	0.585	< 0.001
PK		0.535 ± 1.849a	0.147 ± 0.028a	0.599	< 0.001
OPT		0.655 ± 1.647a	0.127 ± 0.025b	0.584	< 0.001
CRU		0.606 ± 1.698a	0.132 ± 0.026b	0.589	< 0.001
DMS		0.574 ± 1.196a	0.108 ± 0.018c	0.658	< 0.001
STR		0.423 ± 1.128a	0.095 ± 0.017d	0.627	< 0.001

Means within a column followed by the same letter are not significantly different at p< 0.05 level.

The leachate amount was considerably higher in the maize season than in the wheat season, and it differed significantly among N management practices (**Table 2**). PK treatment had the lowest leachate amounts in both the maize and wheat seasons. Compared to FP, STR enhanced soil water infiltration most, followed by DMS treatment. However, OPT and CRU showed limited effects on leachate reduction. CRU treatment had a slightly lower leachate amount than FP only at the beginning of the rotation.

### Runoff and leachate N

**Figure 1:**
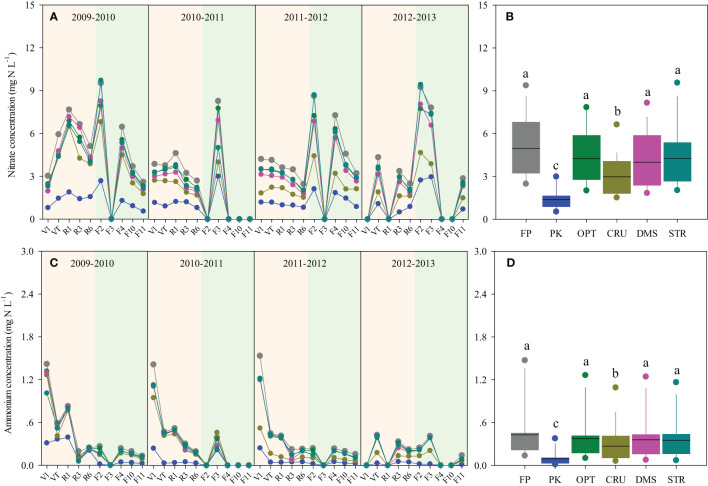
Dynamics of NO_3_^−^-N **(A)** and NH_4_^+^-N **(C)** concentrations in runoff during maize (in the yellow-pink background) and wheat seasons (in the yellow-green background) and the seasonal averages **(B**, **D)** under different N management practices. Box-whisker showed the average, 25th, 50th, and 75th percentiles. Boxes with the same letter indicated that the averages were not significantly different at the *p*< 0.05 level.

The seasonal runoff N concentration dynamics are shown in **Figure 1**. The runoff NO_3_^−^-N concentration was generally higher than NH_4_^+^-N. Averaged across eight seasons, PK treatment was the lowest in runoff NO_3_^−^-N and NH_4_^+^-N concentrations (1.37 mg N L^−1^ and 0.09 mg N L^−1^, respectively, **Figures 1B, D**). The second-lowest runoff N concentration was observed in CRU treatment, with average NO_3_^−^-N and NH_4_^+^-N concentrations of 2.97 mg N L^−1^ and 0.27 mg N L^−1^, respectively. FP, OPT, DMS, and SRT did not vary in runoff N concentrations, but they were higher than PK and CRU treatments (*p*< 0.05).

**Figure 2:**
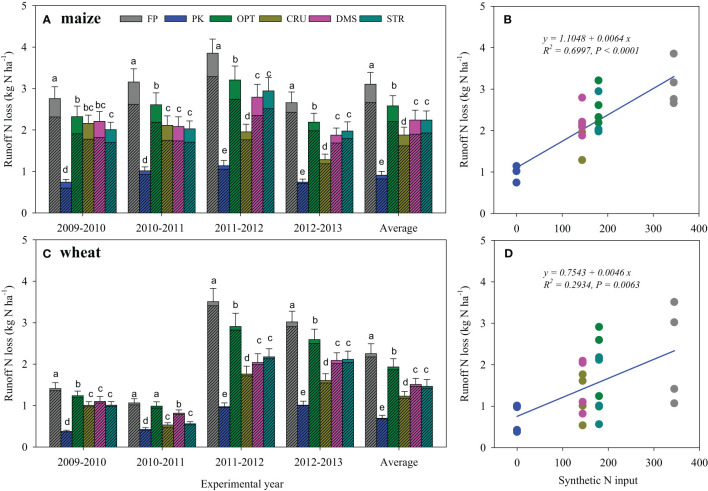
Runoff N losses during maize **(A)** and wheat seasons **(C)** under different N management practices and their relations with synthetic N inputs **(B**, **D)**. Bars in the same season, followed by the same letter, indicated the means were not significantly different at the *p*< 0.05 level. Bars with and without slashes were NO_3_^−^-N and NH_4_^+^-N losses, respectively.

N omission in PK treatment led to the lowest runoff N losses compared to other treatments receiving N (*p*< 0.05). FP resulted in the highest runoff N losses, which were on average 3.11 kg N ha^−1^ and 2.17 kg N ha^−1^ in maize and wheat seasons (**Figures 2A, C**). CRU treatment was most effective in reducing runoff N loss, and it had 39.2% and 45.7% less N loss than FP in maize and wheat seasons (*p<* 0.05). DMS and STR treatments had similar effects, and they had on average 28.7% and 27.7% less runoff N loss in maize season and 32.7% and 34.6% less in wheat season, as compared to FP. Moreover, to a lesser magnitude, OPT treatment also decreased runoff N losses compared to FP (by 17.2% and 14.2% in maize and wheat seasons, respectively). The regression analysis demonstrates linear increases in runoff N losses with increasing synthetic N inputs for both maize (**Figure 2B**, *p*< 0.0001) and wheat seasons (**Figure 2D**, *p*< 0.01).

**Figure 3:**
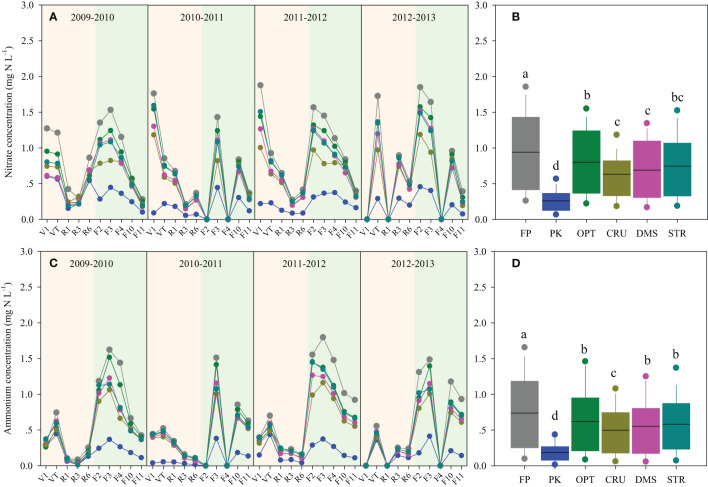
Dynamics of NO_3_^−^-N **(A)** and NH_4_^+^-N **(C)** concentrations in leachate during maize (in the yellow-pink background) and wheat seasons (in the yellow-green background) and the seasonal averages **(B**, **D)** under different N management practices. Box-whisker showed the average, 25th, 50th, and 75th percentiles. Boxes with the same letter indicated that the averages were not significantly different at the *p*< 0.05 level.

The dynamics of leachate N concentrations are shown in **Figure 3**. The NO_3_^−^-N concentration in leachate was slightly higher than that of NH_4_^+^-N. N management practices significantly affected leachate N concentrations. Averaged across eight seasons monitored, the lowest leachate NO_3_^−^-N and NH_4_^+^-N concentrations were recorded in PK, while the greatest was in FP treatment. CRU was the most effective measure to lower leachate N concentration, with average declines of 33.0% and 32.4% in NO_3_^−^-N and NH_4_^+^-N, as compared to FP. DMS was the second most effective, and it reduced NO_3_^−^-N and NH_4_^+^-N concentrations by 26.7% and 24.9%, respectively. Moreover, the leachate NO_3_^−^-N concentrations in OPT and STR were 15.0% and 20.8% lower, and NH_4_^+^-N concentrations were 16.1% and 21.1% lower relative to FP treatment.

**Figure 4:**
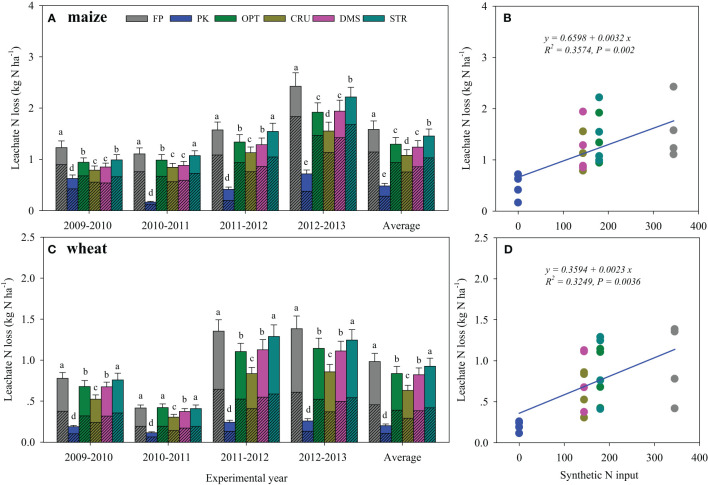
Leachate N losses during maize **(A)** and wheat seasons **(C)** under different N management practices and their relations with synthetic N inputs **(B**, **D)**. Bars in the same season, followed by the same letter, indicated the means were not significantly different at the *p*< 0.05 level. Bars with and without slashes were NO_3_^−^-N and NH_4_^+^-N losses, respectively.

More leachate N loss occurred in maize than in wheat seasons (**Figure 4**). The leachate N was lost mainly as NO_3_^−^-N in maize seasons, but the leachate loads of NH_4_^+^-N and NO_3_^−^-N were comparable in wheat seasons. In both maize and wheat seasons, the lowest and highest leachate N losses were consistently observed in PK and FP treatments, respectively. Compared to FP, adjusted N management practices significantly mitigated leachate N losses. CRU treatment was the most effective, and it reduced leachate N losses by 34.2% and 36.3% in maize and wheat seasons, respectively. OPT and DMS treatments showed similar effects, and they decreased average leachate N losses by 18.1% and 25.3% in maize and 14.3% and 15.8% in wheat seasons, as compared to FP. STR was less effective and declined average leachate N loss only in maize seasons (10.2%, *p*< 0.05).

### Soil nitrate residual

**Figure 5:**
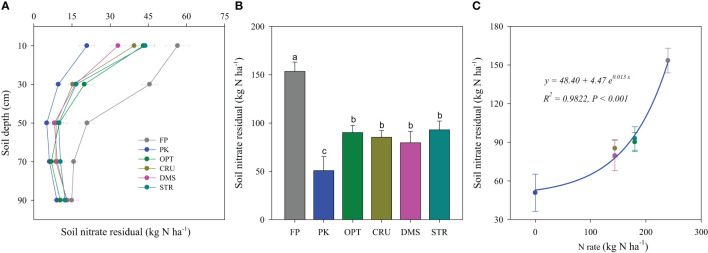
Soil nitrate residual distribution **(A)** and accumulation **(B)** in the 0–90-cm profile under different N management practices and their relation to the N rate **(C)**. Bars, followed by the same letter, indicated the means were not significantly different at the *p*< 0.05 level.

N management practices resulted in significant variations in soil nitrate residuals in the 0–90-cm layer after the wheat harvest in 2013 (**Figure 5**), and the difference decreased with increasing soil depth. PK treatment had the lowest nitrate residual in each soil layer, while FP treatment was constantly the highest (**Figure 5A**). The nitrate accumulation in the 0–90-cm layer was 50.8 kg N ha^−1^ in the PK treatment and increased significantly up to 153.5 kg N ha^−1^ in FP treatment. The adjusted N practices did not vary in nitrate accumulation in the 0–90-cm soil layer (ranging from 79.6 to 92.9 N ha^−1^) but were 41.2%, 44.4%, 48.2%, and 39.5% lower than that in FP treatment (*p*< 0.05).

### Crop yield and NUE

N management practices significantly influenced maize and wheat grain yields (**Table 4**). N omission in PK treatment reduced grain yields, with average declines of 34.8% in maize and 80.1% in wheat as compared to FP treatment. STR treatment produced on average 7.3% more maize yield than FP. OPT and CRU showed significantly higher maize yield than FP in the last two seasons, and the average increments were 5.5% and 4.4% over four seasons. DMS treatment, in general, did not vary with FP in maize yield, except for a slight increase in 2011. Moreover, OPT, STR, and DMS increased wheat yields in the last two seasons. Averaged over four seasons, they produced 6.2%, 3.2%, and 2.7% greater wheat yields than FP. CRU treatment did not differ from FP in wheat grain yield, although it had a reduced N input.

**Table 4:** Crop yield and NUE during maize and wheat seasons under different N management practices.

Treatment	2009–2010	2010–2011	2011–2012	2012–2013	Average yield	2009–2010	2010–2011	2011–2012	2012–2013	Average NUE
	Maize grain yield (kg ha^−1^)					NUE in maize (%)				
FP	8,734a	10,643b	6,366c	8,720b	8,616c	18.6c	30.0c	8.5c	11.2d	17.1d
PK	6,085b	4,895c	4,736d	6,765c	5,620d					
OPT	9,403a	10,267b	7,247a	9,441a	9,090b	34.7b	53.7b	25.1a	26.8b	35.1b
CRU	9,249a	10,192b	6,916ab	9,623a	8,995b	44.5a	66.2a	27.3a	35.7a	43.4a
DMS	9,048a	9,803b	6,763b	8,570b	8,546c	34.4b	49.1b	20.3b	18.1c	30.4c
STR	8,865a	11,234a	7,542a	9,334a	9,244a	31.6b	63.4a	28.1a	25.7b	37.2b
	Wheat grain yield (kg ha^−1^)					NUE in wheat (%)				
FP	7,140a	6,384b	5,488b	3,610c	5,656b	30.8c	27.4d	23.2b	14.5c	24.0c
PK	1,233c	1,421c	1,036c	823d	1,128c	–	–	–	–	–
OPT	6,317b	6,740a	6,454a	4,510a	6,005a	50.8b	50.2b	54.2a	36.9b	48.8b
CRU	6,230b	6,360b	5,706b	4,074b	5,593b	62.5a	59.2a	53.1a	40.6a	53.9a
DMS	6,167b	6,307b	6,250a	4,500a	5,806a	49.3b	44.7b	52.1a	36.8b	45.7b
STR	6,264b	6,192b	6,364a	4,525a	5,836a	50.3b	45.7b	53.3a	37.0b	46.6b

FP, farmers’ fertilization practice; PK, P and K fertilizers only; OPT, optimized NPK fertilization; CRU, control-release N fertilization; DMS, optimized NPK fertilization with 20% of the total nutrients replaced by inputs from duck manure; STR, optimized NPK fertilization plus straw covering. Means within a column followed by the same letter are not significantly different at p< 0.05 level, LSD.

N management practices led to significant variations in crop NUE (**Table 4**). N overapplication in FP treatment led to the lowest NUE, i.e., only 17.1% and 24.0% in maize and wheat, respectively. In contrast, adjusted N management practices greatly enhanced crop NUE. CRU treatment resulted in the most efficient N use, showing an average NUE of 43.4% and 53.9% for maize and wheat, followed by STR and OPT, while, to a lesser magnitude, DMS also achieved higher NUE than FP treatment.

## Discussion

### Runoff and leachate N losses

Agricultural practices greatly impact soil nutrient losses, which may vary as a result of fertilization regimes, tillage, and weather conditions (Dai et al., 2016; Wei et al., 2021). High soil N accumulation was prone to being lost through hydrological pathways when there was rainfall or irrigation (Wang et al., 2010; Liu et al., 2017; Zhang et al., 2021a). In the present study, runoff and leachate amounts were regulated by N management practices that differed in soil disturbance, as these tended to change the sensibility of surface runoff to rainfall. The runoff in PK treatment was the most sensitive to rainfall due to poor plant growth and a lack of soil surface disturbance (e.g., no topdressing) that retained the soil compacted with low impedance to surface water flow. STR and DMS significantly reduced runoff and its sensibility to rainfall but increased leachate. This could be explained by the fact that straw covering and organic matter incorporation increased the resistance to surface water flow and promoted water retention and infiltration due to increased soil porosity and reduced bulk density (Liu et al., 2012; Wang et al., 2019).

Many studies focused on identifying the optimal N fertilizer rates for high crop yield with reduced runoff and leaching N losses (Yang et al., 2017; Zhang et al., 2021a; Zhang et al., 2021b). Our results demonstrated that runoff and leachate N losses were significantly and positively correlated with synthetic N input (**Figures 2**, **4**), indicating it was one of the dominant factors affecting hydrological N losses. FP practice resulted in the greatest N losses via hydrological pathways (**Figures 2**, **4**). The continuous over-application of N fertilizer in FP treatment would drive the soil into an N-saturated condition, resulting in poor N retention (Ju, 2014). By contrast, the reduced N and balanced P and K use in OPT facilitated crop N uptake and considerably decreased runoff and leaching N losses in both maize and wheat seasons.

Hydrological N losses were also regulated by N types and management practices. In comparison to OPT, STR and DMS treatments further reduced runoff N discharges (**Figure 2**), but they sustained or slightly increased N leaching. These results were probably associated with modified soil physical properties (e.g., improved soil porosity and decreased bulk density) following organic matter incorporation, which reduced surface flow but increased water infiltration (Liu et al., 2012; Wang et al., 2019). Moreover, CRU was proven to be the most effective in alleviating hydrological N losses. Regulation of N release through amendment with CRU enhanced crop N uptake (**Table 4**), leaving less soil N to be lost with rainfall.

The magnitude of hydrologic N losses in this study was attributed more to the N concentration than the amount of discharge, as evidenced by the much higher variations in the former (**Table 2**; **Figures 1**, **3**). In line with previous findings (Zhang et al., 2021b), NO_3_^−^-N was obviously dominant over NH_4_^+^-N in inorganic N losses via runoff in the present study (**Figure 1**). Moreover, 70% of the inorganic N discharged in leachate was in the form of NO_3_^−^-N in maize seasons, but there were similar fractions of NO_3_^−^-N and NH_4_^+^-N loads lost to leachate in wheat seasons. This might be due to the low soil temperature limiting the nitrification process (Rodríguez et al., 2005), leading to an increased NH_4_^+^-N concentration in percolation water during the early stages of wheat growth (**Figure 3C**).

### Soil nitrate residual

Excessive N application has resulted in high soil nitrate residuals in cereal and vegetable cropping systems (Cui et al., 2010; Zhang et al., 2021a), leading to an increased risk of N loss and lack of yield response to applied N (Liu et al., 2017; Zhang et al., 2021a). In the current study, FP treatment resulted in a huge soil nitrate accumulation of 153.5 kg N ha^−1^ in the 0–90-cm profile after the last wheat season (**Figure 5**). It was much higher than the acceptable soil nitrate-N level of 90 kg N ha^−1^, as suggested for winter wheat–summer maize rotation fields of North China Plain (Cui et al., 2008a; Cui et al., 2008b). These results indicated the high N rate in FP must be reasonably reduced with the large indigenous soil N being carefully considered. In contrast, adjusted N management practices significantly decreased soil nitrate accumulation to 79.6–92.9 N ha^−1^, suggesting that these practices were generally effective in controlling soil nitrate residual.

### Crop yield and NUE

Improving crop yield and NUE simultaneously is vital to sustainable agricultural production. N fertilization practices have to be managed to match crop N demand in terms of N rate, source, timing, and space (Cui et al., 2010). In the present study, N over-application in FP treatment did not benefit crop yield. Rather, it led to the lowest NUE (averaged 17.1% and 24.0% in maize and wheat, respectively, **Table 4**) at the cost of potential P and K limitations. By contrast, OPT and STR treatments with a reduced N rate of 180 kg N ha^−1^ crop^−1^ and balanced use of P and K facilitated crop N uptake and enhanced crop yield and NUE. Moreover, the positive effects of STR may also result from the more favorable soil conditions created with straw covering, e.g., improved soil temperature status and reduced water loss by evaporation (Chen et al., 2015; Liu et al., 2017). Corroborating our results, a regionwide optimal N rate of 150–180 kg N ha^−1^ crop^−1^ has been recommended for the maize–wheat rotation systems in North China (Cui et al., 2008b; Zhang et al., 2011). These results suggest that a reduced N rate of 180 kg N ha^−1^ crop^−1^ is sufficient, and the combination with straw covering is one measure suitable for the studied region in terms of crop yield and NUE improvement.

Several studies indicated improved cereal yield and NUE by co-applicating organic and synthetic N fertilizers relative to synthetic N alone (Das and Adhya, 2014; Wei et al., 2021), as the mineral N matched better with crop N demand. In the present study, however, DMS treatment appeared to be less effective than OPT in boosting crop yield and NUE. The discrepancy in results might be associated with differences in organic fertilizers and cropping systems. In our study, the organic matter incorporation with manure may have induced N immobilization and reduced the size of available N pools, particularly in the maize season with relatively high soil moisture and short growth duration (Devevre and Horwath, 2000; Said-Pullicino et al., 2014). Regulation of N release through amendment with CRU greatly improved crop NUE. Meanwhile, it increased maize yield and sustained a similar wheat yield to FP with reduced N input. Overall, our results suggest that CRU could be used as a feasible and effective approach to improve crop NUE and grain yield under maize-wheat rotation, provided that it is applied in the right type and rate according to crop N demand.

### Implication and perspectives

During the past decades, the intensification of agricultural production has played a crucial role in nourishing the livelihood of the growing population (Li et al., 2017). However, excessive input of synthetic N also brought great challenges to agricultural sustainability (Zhang et al., 2015). A package of rational N management practices needs to be integrated to achieve the dual goals of ensuring food security and mitigating environmental costs (Chen et al., 2014). The present study illustrated that the adjusted N management practices significantly promoted crop NUE, increased or at least sustained crop yield, and reduced hydrological N losses and soil nitrate N residual, which is essential for agricultural sustainability. While we are encouraged by the great potential of these approaches, we need to know the limitations of this study. The field investigations were conducted in a lakeshore agricultural area, and the main concerns were the hydrological N losses. The other N pathways, such as gas N emissions, should be explored. More experiments and model-based studies are warranted to achieve a more comprehensive understanding of the interactions of soil C, N, and water processes and balances in the agriculture system.

## Conclusion

Adjusted N management practices significantly enhanced crop NUE and improved grain yield, but the magnitude of these benefits varied with N rates and types used. The optimized N rate with or without straw returning achieved the highest crop yield. Regulation of N release through amendment with CRU was the most effective in fertilizer N use and mitigation of hydrological N loss. Moreover, organic matter incorporation in STR and DMS treatments further reduced runoff N discharges than OPT, but they sustained or slightly increased N leaching. Excessive N application in FP resulted in considerable nitrate accumulation in the 0–90-cm soil profile. The adjusted N management practices effectively controlled those close to the acceptable soil nitrate-N level. Overall, our results suggest that efforts using optimized N treatment integrated with CRU or straw returning might be feasible for sustainable crop production in this region.

## Data availability statement

The original contributions presented in the study are included in the article/**Supplementary Material**. Further inquiries can be directed to the corresponding authors.

## Author contributions

LW: Conceptualization, Data curation, Investigation, Methodology, Writing – original draft. LM: Data curation, Writing – review & editing. YL: Software, Validation, Writing – review & editing. C-MG: Writing – review & editing. JW: Investigation, Writing – review & editing. FZ: Investigation, Writing – review & editing. ZL: Supervision, Writing – review & editing. DT: Software, Validation, Writing – review & editing.
