# Supplementary material for: Managing nitrogen for sustainable crop production with reduced hydrological nitrogen losses under a winter wheat–summer maize rotation system: an eight-season field study
Source: Front Plant Sci. 2023 Nov 14;14:1274943. doi: 10.3389/fpls.2023.1274943 (PMC10682078; doi:10.3389/fpls.2023.1274943)
Supplement: Supplementary file 1 [file DataSheet_1.docx]

Supplementary Material

**Supplementary Figure 1.** Daily maximum, minimum air temperature, and rainfall during the study period.


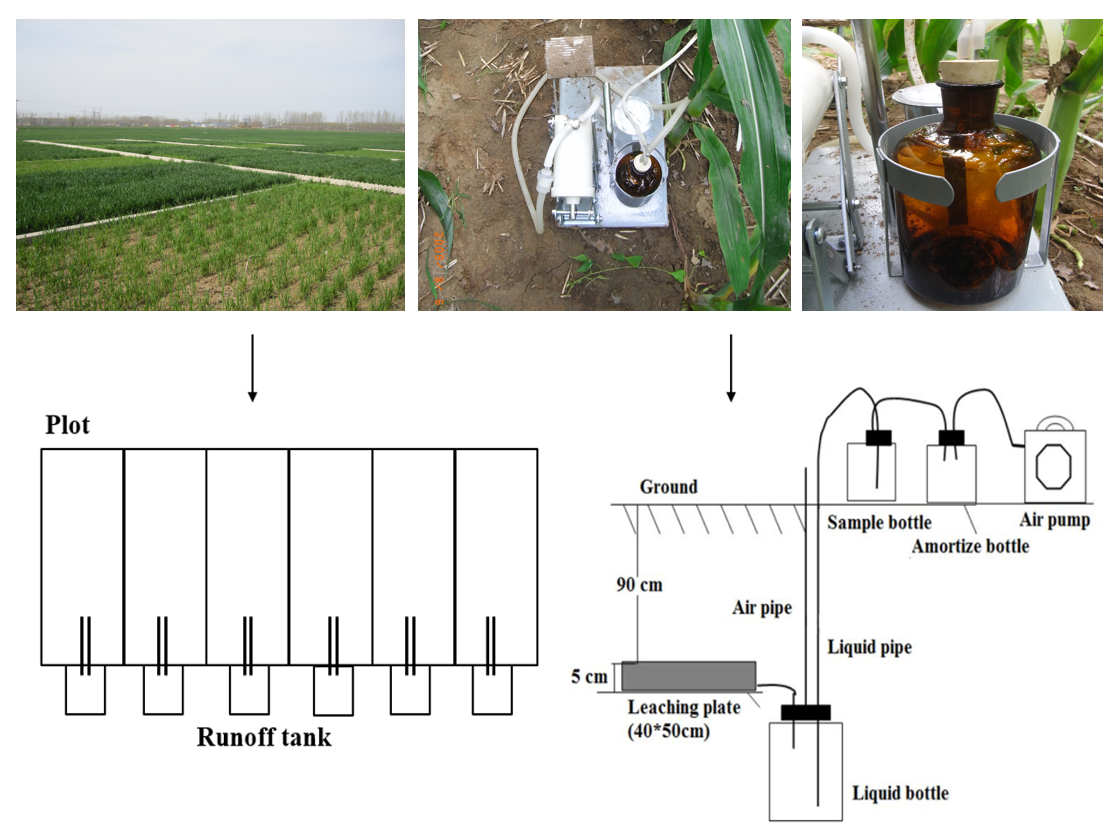


**Supplementary Figure 2.** Illustration of leachate and runoff collection.

**Supplementary Figure 3.** Cumulative runoff amount during maize, wheat season under different N management practices.

**Supplementary Figure 4.** Cumulative leachate amount during maize, wheat season under different N management practices.
